# Optimization of DNA Extraction from Field-Collected Mammalian Whole Blood on Filter Paper for *Trypanosoma cruzi* (Chagas Disease) Detection

**DOI:** 10.3390/pathogens10081040

**Published:** 2021-08-17

**Authors:** Bonnie E. Gulas-Wroblewski, Rebecca B. Kairis, Rodion Gorchakov, Anna Wheless, Kristy O. Murray

**Affiliations:** 1 Department of Pediatrics, Section of Pediatric Tropical Medicine, National School of Tropical Medicine, Baylor College of Medicine and Texas Children’s Hospital, Houston, TX 77030, USA; bonnie.gulas@ag.tamu.edu (B.E.G.-W.); Rebecca.b.kairis@uth.tmc.edu (R.B.K.); rodion.gorchakov@kaust.edu.sa (R.G.); awheless@unc.edu (A.W.); 2 Texas A&M Natural Resources Institute, College Station, TX 77843, USA; 3 The William T. Shearer Center for Human Immunobiology, Texas Children’s Hospital, Houston, TX 77030, USA; 4 The University of Texas Health Science Center at Houston, Houston, TX 77030, USA; 5 Health, Safety and Environment Department, King Abdullah University of Science and Technology, Thuwal 23955, Saudi Arabia; 6 Department of Biochemistry and Biophysics, University of North Carolina at Chapel Hill, Chapel Hill, NC 27599, USA

**Keywords:** blood filter paper, Chagas disease, DNA extraction, Nobuto strip, *Trypanosoma cruzi*, mammalian surveillance, neglected tropical diseases, PCR

## Abstract

Blood filter paper strips are cost-effective materials used to store body fluid specimens under challenging field conditions, extending the reach of zoonotic pathogen surveillance and research. We describe an optimized procedure for the extraction of parasite DNA from whole blood (WB) stored on Type I Advantec Nobuto strips from both experimentally spiked and field-collected specimens from canine and skunks, respectively. When comparing two commercial kits for extraction, Qiagen’s DNeasy Blood & Tissue Kit performed best for the detection of parasite DNA by PCR from *Trypanosoma cruzi*-spiked canine WB samples on Nobuto strips. To further optimize recovery of β-actin from field-collected skunk WB archived on Nobuto strips, we modified the extraction procedures for the Qiagen kit with a 90 °C incubation step and extended incubation post-addition of proteinase K, a method subsequently employed to identify a *T*. *cruzi* infection in one of the skunks. Using this optimized extraction method can efficaciously increase the accuracy and precision of future molecular epidemiologic investigations targeting neglected tropical diseases in field-collected WB specimens on filter strips.

## 1. Introduction

The parasitic protozoan *Trypanosoma cruzi* is the etiologic agent of Chagas disease, which is maintained in domestic, peridomestic, and sylvatic transmission cycles by a diversity of triatomine vectors and mammalian hosts [1]. This neglected tropical parasite infects an estimated 6–7 million people across the Americas, making the zoonosis one of the most significant in terms of disease burden and public health importance in the western hemisphere [2]. Chagas disease and other neglected tropical diseases (NTDs) are particularly devastating for impoverished populations in remote regions with limited public health infrastructure [3,4,5,6]. In addition, the zoonotic nature of NTDs necessitates their investigation and control within a One Health framework, which holistically integrates domestic animal, wildlife, environmental, ecological, and public health [7,8]. Consequently, public health workers and epidemiologists face costly and logistical challenges in not only adequately sampling human and animal populations, but also preserving biological specimens of high enough quality for genomic applications under adverse field conditions and for prolonged periods of time [9,10]. However, NTD research and elimination programs are relatively underfunded, especially in comparison to those targeting pandemic-producing pathogens, exacerbating the challenges of NTD field-to-laboratory workflows [10,11,12]. In order to facilitate an inexpensive, reliable, and sensitive method option for polymerase chain reaction (PCR)-based NTD epidemiologic surveillance, we evaluated the performance of three commercial extraction kits with adjusted DNA extraction protocols for the recovery of *T*. *cruzi* DNA from canine whole blood (WB) preserved on filter paper. We then validated the optimized procedure via PCR recovery of β-actin from WB specimens collected from skunks (Mammalia: Mephitidae) and archived on filter papers. Developing optimized protocols for *T*. *cruzi* DNA extraction from blood filter papers greatly expands the efficiency and effectiveness of field investigations into the molecular epidemiology and surveillance of Chagas disease among mammalian reservoirs.

## 2. Results

### 2.1. Optimization with T. cruzi-Spiked Canine WB Samples

**Figure 1:**
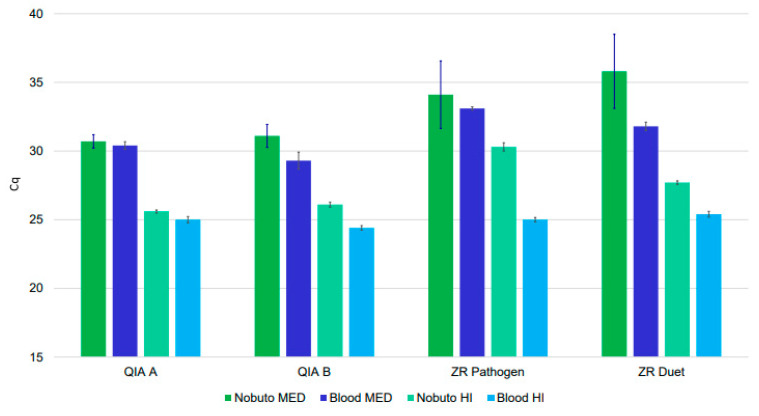
*Trypanosoma cruzi* DNA recovery from spiked canine whole blood specimens. Bars indicate one standard deviation. As detailed in the text for use with Qiagen DNeasy Blood & Tissue Kit: QIA A = extraction optimization method A; QIA B = extraction optimization method B. ZR Pathogen = Zymo Research Quick-DNA/RNA Pathogen Miniprep; ZR Duet = Zymo Research ZR-Duet DNA/RNA Miniprep Plus kit. Cq = quantification cycle; Nobuto = whole blood samples processed from Nobuto blood filter papers; Blood = whole blood samples processed directly; MED = medium spiking load; HI = high spiking load.

*T*. *cruzi* DNA was successfully extracted and recovered via quantitative (q) PCR for the spiked WB samples processed with each extraction method from both the Zymo Research (Zymo Research, Irvine, CA, USA) and Qiagen (Qiagen, Germantown, MD, USA) kits, though each procedure varied in the recovery of target DNA as measured by quantification cycle (Cq) values of the qPCR output (Figure 1, Supplementary Table S1). DNA extraction optimization methods employing the Qiagen DNeasy Blood & Tissue Kit outperformed those relying on Zymo Research kits for both WB and WB stored on Nobuto strips (Supplementary Table S1). At medium spiking loads, the difference between the mean Cq value recovered for WB versus WB-saturated Nobuto strips was substantially lower for extraction optimization method A (0.25) compared to the variances evidenced with extraction optimization method B (1.77), the Quick-DNA/RNA Pathogen Miniprep kit (1.1), and the ZR-Duet DNA/RNA Miniprep Plus kit (3.95). Similarly, differences between the mean Cq values generated by high spiking loads for WB and WB stored on Nobuto blood filter paper were lower for the Qiagen DNeasy Blood & Tissue Kit methods (0.65 for extraction optimization method A and 1.74 for extraction optimization method B) than for either of the Zymo Research kits (2.31 for ZR-Duet DNA/RNA Miniprep Plus kit and 5.22 for the Quick-DNA/RNA Pathogen Miniprep kit) (Supplementary Table S1).

### 2.2. Optimization with Skunk WB Samples

Once we established the enhanced capacity of the Qiagen DNeasy Blood & Tissue Kit to recover *T*. *cruzi* DNA from spiked WB specimens stored on Nobuto strips, we repeated extraction optimization methods A and B with an additional alternate extraction procedure on samples of skunk WB archived on Nobuto blood filter paper (Supplementary Table S2). Since the *T*. *cruzi* status of these animals was unknown, we employed a qPCR assay developed to detect β-actin-encoding DNA from mammals (Table 1) [13].

**Figure 2:**
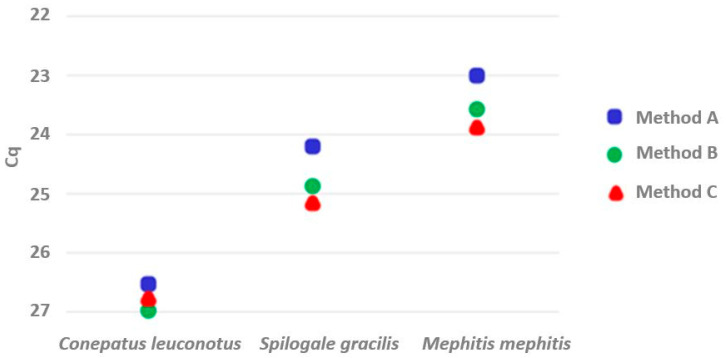
β-actin DNA recovered from skunk whole blood archived on Nobuto blood filter paper strips. DNA extracted according to protocols outlined in the text for Qiagen DNeasy Blood & Tissue Kit: Method A = extraction optimization method A; Method B = extraction optimization method B; Method C = extraction optimization method C. Cq = quantification cycle.

Each of the extraction optimization methods for the Qiagen kit recovered β-actin DNA as indicated by qPCR analysis (Figure 2, Supplementary Table S3). Extraction optimization A outperformed the other methods for all three samples, recovering an average of 60% more DNA than the other two protocols. In the case of relatively higher concentrations of DNA (samples from the western spotted (*Spilogale gracilis*) and striped skunks (*Mephitis mephitis*)), extraction optimization B extracted more DNA than did method C, whereas the latter method excelled with the comparatively lower amount of β-actin DNA present in the American hog-nosed skunk (*Conepatus leuconotus*) sample (Figure 2). Subsequent evaluation of these WB samples for the presence of *T*. *cruzi* DNA was performed using extraction optimization A, identifying parasitic infection in the western spotted skunk (Cq value of 27.8) and not detecting *T*. *cruzi* DNA from either the American hog-nosed or striped skunk samples.

## 3. Discussion

Our objective was to evaluate the most effective method for extracting DNA from WB preserved on blood filter paper, comparing three commonly used extraction kits with three modifications on samples from four mammalian species analyzed for two types of DNA. Our comparison of extraction methodologies using spiked and archived specimens demonstrates that using the Qiagen DNeasy Blood & Tissue Kit with an initial 90 °C incubation step and extended 56 °C incubation step after addition of proteinase K provided the optimal recovery of both β-actin and *T*. *cruzi* DNA from WB stored on Nobuto strips. Incubation for an extended period of time and/or at elevated temperatures during the blood cells lysis and removal steps of DNA extraction from WB can increase the purity of the DNA recovered [15]. Stowell et al. (2018) recovered the most DNA from WB stored on various filter paper types by combining the QIAamp kit, Nobuto strips, and an incubation modification step of post-ATL overnight shaking at 37 °C and a 2-h incubation at 56 °C following application of proteinase K. The authors also report consistently high recovery of DNA with all high incubation modifications they employed on Classic FTA and Nobuto filter papers [16]. By adjusting these conditions within the optimized extraction protocols, we successfully increased the quantity of recovered β-actin and *T*. *cruzi* DNA detectable by qPCR analysis.

We focused our DNA extraction optimization analyses on Nobuto blood sampling filter paper due to this product’s relatively low cost and wide availability through online markets. For instance, Nobuto blood filter paper is currently available through online marketplaces for as low as USD 0.34 per filter strip. The cost-effectiveness and ease with which filter papers can be used to store blood products and other body fluid specimens without pre-preparation or temperature control constraints makes them a convenient collection method for diagnostic sampling in the field, particularly in cases of remote work in warm climates and with geographically-isolated populations [9,10,17]. The ability of filter papers to preserve extractable DNA in WB specimens for multiple years at room temperature further facilitates their application in biobanking and retrospective analyses [10,17]. In practice, DNA stored on filter paper has been extensively employed in population genetics analyses [16,18,19,20,21] and wildlife disease detection [22,23], while its application in anthropocentric epidemiology has been largely limited to serological diagnostic screening, pharmaceutical development, and drug monitoring [9,10,24]. Previous field studies reported the limitation of sample volume available for extractions and the reduced integrity, stability, and purity of extracted DNA as potential factors contributing to the loss of sensitivity of PCR-based investigations using blood filter papers [9,10,25]. By increasing the yield of PCR-detectable parasite DNA from WB archived on Nobuto strips, our optimized extraction protocol advances the ease and reliability of using this relatively inexpensive method in field-to-laboratory epidemiological surveillance programs.

Stringent validation guidelines would mandate the use of blood samples from a greater range of mammalian species than our study encompasses to assess the potential influence of matrix heterogeneity on the DNA extraction yield. However, we addressed the putative influence of matrix heterogeneity on DNA recovery indirectly via testing WB collected from a domestic dog and three skunk species. Furthermore, a DNA extraction optimization study using samples of blood from domestic dogs, elk (*Cervus elaphus*), bighorn sheep (*Ovis canadensis*), and mule deer (*Odocoileus hemionus*) did not detect any variations in DNA yield between the taxa when performing extractions from multiple types of filter paper [16]. Blood matrix properties that affect DNA recovery most likely differ at higher phylogenetic levels [16]. Future studies, such as those requiring stringent validation for clinical research purposes or those surveying non-human animals, would benefit from repeating our optimization experiments on samples from several individuals of the same species and/or on a wider variety of species, particularly those from other Classes (e.g., avians, reptiles, amphibians).

Future research would also benefit from evaluating the performance of our optimized extraction protocol for the recovery of DNA from other body fluids and biological samples stored on blood filter paper. In addition to elevating the ease and inclusivity of sampling via non-invasive means of collection, the use of non-WB specimens holds great potential for expansion of NTD surveillance across different stages of disease progression [26,27,28]. The utility of filter paper for the storage of a diversity of biological samples is illustrated by its use to preserve DNA from manta ray (*Manta birostris*) mucus [19] and human buccal cells [29]. We expect our extended incubation steps to especially elevate DNA yield from urine, semen, breast milk, and other body fluids with complex specimen matrices [28,30].

## 4. Materials and Methods

### 4.1. Samples

All WB samples were collected on Type I Advantec^®^ (Tokyo, Japan) Nobuto blood sampling filter paper. Canine WB from a female German Shepherd domestic dog was scavenged from excess WB collected for clinical purposes and donated by a local veterinary hospital (Houston, TX, USA). Skunk WB samples were collected from an American hog-nosed skunk, a striped skunk, and a western spotted skunk as part of a biobanking project conducted by Angelo State Natural History Collections in the Department of Biology, Angelo State University, San Angelo, TX (Supplementary Table S2). *T*. *cruzi* parasites used for spiking were the Vero cell culture supernatants of the parasite strain TD25 isolated from a triatomine collected in Texas [31].

### 4.2. Canine WB Samples Spiked with T. cruzi

Fresh WB from a *T*. *cruzi*-negative domestic dog was spiked with *T*. *cruzi* parasites cultured on Vero cells. WB was prepared in 1 mL aliquots accordingly: one unspiked control and duplicates for each of the *T*. *cruzi* spiking loads, medium (MED; expected cycle threshold (Cq = 29–30) and high (HI; expected Cq = 24–25) spiked with 20 µL of the respective dilution of parasite. The medium spiking load (MED) used in our experiments corresponds to Cq values at the lower end of the assay’s dynamic range [8]. Medium and high spiking loads were confirmed through controls combining 20 µL of the respective dilution of parasite with 1 mL of phosphate-buffered saline solution. Spiked and control samples were processed in duplicate either directly as WB (50 µL) or post-application to Nobuto blood filter strips. In the latter case, 50 µL of each treatment of WB was applied to Nobuto strips and thoroughly dried in a biosafety cabinet for at least 30 min to replicate field preparation of filter paper specimens. Each Nobuto strip was cut into four, equally-sized pieces that were combined with ATL lysis buffer in a 1.5 mL tube for further processing. One aliquot was processed directly without spiking in order to confirm the negative *T*. *cruzi* status of the canine patient.

For DNA extractions, we tested three extraction kits to determine optimized methods for extracting *T*. *cruzi* DNA from the spiked canine WB samples. We followed manufacturer instructions for DNA extraction from WB for the Quick-DNA/RNA Pathogen Miniprep and ZR-Duet DNA/RNA Miniprep Plus kits. Two alternate DNA extraction optimizations were assessed for the DNeasy Blood & Tissue Kit. In extraction optimization method A for the Qiagen kit, samples were mixed with 150 µL or 180 µL of ATL for direct WB or Nobuto strips, respectively, and incubated at 90 °C for 15 min. Following this incubation period, 20 µL of proteinase K was added and the sample was incubated at 56 °C for 60 min. Next, we added 200 µL of AL buffer, incubated the sample for 10 min at 56 °C, vortexed and spun the sample at maximum speed for 1 min, collected the supernatant, and proceeded to follow manufacturer instructions. Extraction optimization Method B resembled extraction optimization method A with the exclusion of the initial 15 min 90 °C incubation period and the reduction of the 56 °C incubation period post-addition of proteinase K from 60 min to 15 min.

For each evaluation of sample DNA content, five microliters of extracted DNA were run in duplicate in a 20-µL reaction with TaqMan Fast Advanced Master Mix (Thermo Fisher Scientific, Waltham, MA, USA) on a ViiA 7 Real Time PCR System (Thermo Fisher Scientific). Duplicate extraction negative controls, extraction positive controls, and no template controls were included in each qPCR analysis. Mean DNA recovery estimates were calculated from the average Cq values from the duplicates of each sample tested.

Detection of *T*. *cruzi* was performed using primers Cruzi 1 and Cruzi 2 and the probe Cruzi 3 described by Piron et al. (2007) [14], which amplifies a fragment of 166 base pairs (bp) in the satellite DNA of all *T*. *cruzi* lineages (Table 1). The assay exhibits a specificity of 100% and a four-log dynamic range with its lower end positioned at 10 parasites/mL of blood corresponding to Cq values about 30 [14].

### 4.3. DNA Extraction from Skunk WB Samples

In preparation for DNA extraction, the biobanked skunk Nobuto strips were processed to ensure equivalent quantities of WB were sampled per extraction replicate from each strip. One section approximately 5 × 5 mm in length was cut from each WB-saturated Nobuto strip and further divided into four, equally-sized pieces that were transferred into a 1.5 mL tube with ATL lysis buffer for continued processing. DNA was extracted from each strip in accordance with extraction optimization A and B as described above. In addition, we evaluated a third extraction optimization with the Qiagen DNeasy Blood & Tissue Kit for each of these archived WB specimens. Extraction optimization C replicates the steps of extraction optimization B with extension of the 56 °C incubation period following the addition of proteinase K from 15 min to 16 h. The DNA extracted from each treatment was then run in duplicate on qPCR with negative and positive controls.

## 5. Conclusions

Optimization of DNA extraction from WB preserved on blood filter papers can extend the reach, efficacy, and reliability of infectious disease research and surveillance, particularly for investigations in which field constraints typically limit the capacity for body fluid collection and preservation. Developing these procedures for use with commercially-available and user-friendly kits and Nobuto strips further enhances their cost-efficiency and simplicity of application to molecular epidemiological studies. We identify one such optimized DNA extraction method: the Qiagen DNeasy Blood & Tissue Kit with the addition of an initial 90 °C incubation step and extended, post-proteinase K 56 °C incubation step, which provided the most accurate and precise recovery of *T*. *cruzi* DNA. Future work should adopt and continuously adapt this protocol to maximize the sensitivity of these techniques across a range of storage and extraction products, body fluid specimens, target pathogens, and host species sampled.

## Figures and Tables

**Table 1:** Primer and probe sets used in qPCR analysis for detection of *Trypanosoma cruzi* DNA and β-actin. Cruzi TaqMan assay developed by Piron et al. (2007) [14]. β-actin TaqMan assay developed by Piorkowski et al. (2014) [13].

TaqMan Assay	Primer	Sequence (5′–3′)	Probe	Sequence (5′–3′)
*T*. *cruzi*	Cruzi 1	ASTCGGCTGATCGTTTTCGA	Cruzi 3	CACACACTGGACACCAA
	Cruzi 2	AATTCCTCCAGCAGCGGATA		
β-actin	Act. f	GTSTGGATYGGHGGHTCBATC	Act. p	ACCTTCCAGCAGATGTGGATC
	Act. r	GAYTCRTCRTAYTCCTSCTTG		

## Supplementary Materials

The following are available online at https://www.mdpi.com/article/10.3390/pathogens10081040/s1, Table S1: Quantitative PCR results for *Trypanosoma cruzi* assays performed on spiked canine whole blood specimens; Table S2: Collection information pertaining to skunk whole blood samples used in DNA extraction optimization testing; Table S3: Quantitative PCR results for β-actin assays performed on skunk whole blood samples archived on Nobuto blood filter strips and processed using three optimized Qiagen DNeasy Blood & Tissue Kit DNA extraction methods as detailed in text.

## References

[1] Bern C., Messenger L.A., Whitman J.D., Maguire J.H. (2019). Chagas Disease in the United States: A Public Health Approach. Clin. Microbiol. Rev..

[2] World Health Organization (2020). Chagas Disease (American Trypanosomiasis), Fact Sheet No. 340. https://www.who.int/en/news-room/fact-sheets/detail/chagas-disease-(american-trypanosomiasis).

[3] Hanford E.J., Zhan F.B., Lu Y., Giordano A. (2007). Chagas disease in Texas: Recognizing the significance and implications of evidence in the literature. Soc. Sci. Med..

[4] Sarkar S., Strutz S.E., Frank D.M., Rivaldi C.L., Sissel B., Sánchez-Cordero V. (2010). Chagas disease risk in Texas. PLoS Negl. Trop. Dis..

[5] Hotez P.J., Bottazzi M.E., Dumonteil E., Valenzuela J.G., Kamhawi S., Ortega J., Rosales S.P.D.L., Cravioto M.B., Tapia-Conyer R. (2012). Texas and Mexico: Sharing a Legacy of Poverty and Neglected Tropical Diseases. PLoS Negl. Trop. Dis..

[6] Hotez P.J., Dumonteil E., Woc-Colburn L., Serpa J.A., Bezek S., Edwards M.S., Hallmark C.J., Musselwhite L.W., Flink B.J., Bottazzi M.E. (2012). Chagas disease: “The new HIV/AIDS of the Americas”. PLoS Negl. Trop. Dis..

[7] Daszak P., Cunningham A.A., Hyatt A.D. (2000). Emerging infectious diseases of wildlife threats to endangerment. Conserv. Biol..

[8] Cunningham A.A., Daszak P., Wood J.L. (2017). One Health, emerging infectious diseases and wildlife: Two decades of progress?. Philos. Trans. R. Soc. B Biol. Sci..

[9] Bereczky S., Färnert A., Mårtensson A., Gil J.P. (2005). Short report: Rapid dna extraction from archive blood spots on filter paper for genotyping of plasmodium falciparum. Am. J. Trop. Med. Hyg..

[10] Lim M.D. (2018). Dried Blood Spots for Global Health Diagnostics and Surveillance: Opportunities and Challenges. Am. J. Trop. Med. Hyg..

[11] Gyapong J., Gyapong M., Yellu N., Anakwah K., Amofah G., Bockarie M., Adjei S. (2010). Integration of control of neglected tropical diseases into health-care systems: Challenges and opportunities. Lancet.

[12] Ehrenberg J.P., Zhou X.-N., Fontes G., Rocha E.M.M., Tanner M., Utzinger J. (2020). Strategies supporting the prevention and control of neglected tropical diseases during and beyond the COVID-19 pandemic. Infect. Dis. Poverty.

[13] Piorkowski G., Baronti C., de Lamballerie X., de Fabritus L., Bichaud L., Pastorino B., Bessaud M. (2014). Development of generic Taqman PCR and RT-PCR assays for the detection of DNA and mRNA of β-actin-encoding sequences in a wide range of animal species. J. Virol. Methods.

[14] Piron M., Fisa R., Casamitjana N., López-Chejade P., Puig L., Vergés M., Gascon J., Prat J.G.I., Portús M., Sauleda S. (2007). Development of a real-time PCR assay for Trypanosoma cruzi detection in blood samples. Acta Trop..

[15] Qamar W., Khan M.R., Arafah A. (2017). Optimization of conditions to extract high quality DNA for PCR analysis from whole blood using SDS-proteinase K method. Saudi J. Biol. Sci..

[16] Stowell S.M.L., Bentley E.G., Gagne R.B., Gustafson K.D., Rutledge L.Y., Ernest H.B. (2018). Optimal DNA extractions from blood on preservation paper limits conservation genomic but not conservation genetic applications. J. Nat. Conserv..

[17] Michaud V., Gil P., Kwiatek O., Prome S., Dixon L., Romero L., Le Potier M.-F., Arias M., Couacy-Hymann E., Roger F. (2007). Long-term storage at tropical temperature of dried-blood filter papers for detection and genotyping of RNA and DNA viruses by direct PCR. J. Virol. Methods.

[18] Sacks B.N., Brown S.K., Ernest H.B. (2004). Population structure of California coyotes corresponds to habitat-specific breaks and illuminates species history. Mol. Ecol..

[19] Kashiwagi T., Maxwell E.A., Marshall A., Christensen A.B. (2015). Evaluating manta ray mucus as an alternative DNA source for population genetics study: Underwater-sampling, dry-storage and PCR success. PeerJ.

[20] Nunziata S.O., Wallenhorst P., Barrett M.A., Junge R.E., Yoder A.D., Weisrock D.W. (2016). Population and conserva-tion genetics in an Endangered lemur, Indri indri, across three forest reserves in Madagascar. Int. J. Primatol..

[21] Stowell S.M.L., Gagne R.B., McWhirter D., Edwards W., Ernest H.B. (2020). Bighorn sheep genetic structure in Wyoming reflects geography and management. J. Wildl. Manag..

[22] Forzán M., Wood J. (2013). Low detection of ranavirus dna in wild postmetamorphic green frogs, rana (lithobates) clamitans, despite previous or concurrent tadpole mortality. J. Wildl. Dis..

[23] LeClaire S., Menard S., Berry A. (2014). Molecular characterization of Babesia and Cytauxzoon species in wild South-African meerkats. Parasitology.

[24] Lindstrom B., Ericsson O., Alvan G., Rombo L., Ekman L., Rais M., Sjoqvist F. (1985). Determination of chloroquine and its de-sethyl metabolite in whole blood: An application for samples collected in capillary tubes and dried on filter paper. Ther. Drug Monit..

[25] Färnert A., Arez A.P., Correia A.T., Björkman A., Snounou G., Rosário V.D. (1999). Sampling and storage of blood and the detection of malaria parasites by polymerase chain reaction. Trans. R. Soc. Trop. Med. Hyg..

[26] Mfuh K.O., Yunga S.T., Esemu L.F., Bekindaka O.N., Yonga J., Djontu J.C., Mbakop C.D., Taylor D.W., Nerurkar V.R., Leke R.G.F. (2017). Detection of Plasmodium falciparum DNA in saliva samples stored at room temperature: Potential for a non-invasive saliva-based diagnostic test for malaria. Malar. J..

[27] Bezerra G.S.N., Barbosa W.L., da Silva E.D., Leal N.C., Medeiros Z. (2019). Urine as a promising sample for Leishmania DNA extraction in the diagnosis of visceral leishmaniasis—A review. Braz. J. Infect. Dis..

[28] Ronca S.E., Gulas-Wroblewski B.E., Kairis R.B., Murray K.O. (2021). RNA extraction techniques of different body fluids for Zika virus: Blood, genitourinary specimens, saliva, and other relevant fluids. Zika Virus Impact, Diagnosis, Control, and Models.

[29] He H., Argiro L., Dessein H., Chevillard C. (2007). Improved technique that allows the performance of large-scale SNP genotyping on DNA immobilized by FTA^®^ technology. Infect. Genet. Evol..

[30] Gorchakov R., Gulas-Wroblewski B.E., Ronca S.E., Ruff J.C., Nolan M.S., Berry R., Alvarado R.E., Gunter S.M., Murray K.O. (2019). Optimizing PCR Detection of West Nile Virus from Body Fluid Specimens to Delineate Natural History in an Infected Human Cohort. Int. J. Mol. Sci..

[31] Talavera-López C., Messenger L.A., Lewis M.D., Yeo M., Reis-Cunha J.L., Matos G.M., Bartholomeu D.C., Calzada J.E., Saldaña A., Ramírez J.D. (2021). Repeat-Driven Generation of Antigenic Diversity in a Major Human Pathogen, Trypanosoma cruzi. Front. Cell. Infect. Microbiol..

