# Supplementary material for: Optimization of DNA Extraction from Field-Collected Mammalian Whole Blood on Filter Paper for Trypanosoma cruzi (Chagas Disease) Detection
Source: Pathogens. 2021 Aug 17;10(8):1040. doi: 10.3390/pathogens10081040 (PMC8398220; doi:10.3390/pathogens10081040)
Supplement: Supplementary file 1 [file pathogens-10-01040-s001.zip › pathogens-1219823-supplementary.pdf]

Supplementary Table 1. Quantitative PCR results for *Trypanosoma cruzi* assays performed on spiked canine whole blood specimens. DNA extraction optimization methods detailed in text. Ct = cycle threshold; WB Direct = whole blood samples processed directly; WB Nobuto = whole blood samples processed from Nobuto blood filter papers; MED = medium spiking load; HI = high spiking load.

DNA Extraction Method	WB Direct				WB Nobuto			
	MED		HI		MED		HI	
	Mean Ct Values	Standard Deviation	Mean Ct Values	Standard Deviation	Mean Ct Values	Standard Deviation	Mean Ct Values	Standard Deviation
Qiagen Extraction Optimization Method A	30.4	0.28	25.0	0.23	30.7	0.49	25.6	0.11
Qiagen Extraction Optimization Method B	29.3	0.62	24.4	0.17	31.1	0.84	26.1	0.18
Zymo Research Quick-DNA/RNA Pathogen Miniprep	33.1	0.12	25.0	0.16	34.1	2.64	30.3	0.30
Zymo Research ZR-Duet™ DNA/RNA MiniPrep Plus kit	31.8	0.30	25.4	0.21	35.7	2.70	27.7	0.13

Supplementary Table 2. Collection information pertaining to skunk whole blood samples used in DNA extraction optimization testing.

Angelo State Natural History Collections specimen number	Species	Collection location	Collection date
ASK 11844	*Spilogale gracilis*	Tom Green County, Texas, USA	January 7, 2017
ASK 11857	*Mephitis mephitis*	Tom Green County, Texas, USA	February 18, 2014

ASK 11860	*Conepatus leuconotus*	Tom Green County,<br><br>Texas, USA	August 27, 2016

Supplementary Table 3. Quantitative PCR results for β-actin assays performed on skunk whole blood samples archived on Nobuto blood filter strips and processed using three optimized Qiagen DNeasy Blood & Tissue Kit DNA extraction methods as detailed in text. Ct=cycle threshold.

Samples	Mean Ct Values			A/B Fold Difference	A/C fold Difference
	Extraction optimization method A	Extraction optimization method B	Extraction optimization method C		
Conepatus leuconotus, ASK 11860	26.5	27.0	26.8	1.4	1.2
Spilogale gracilis, ASK 11844	24.3	24.9	25.2	1.6	1.9
Mephitis mephitis, ASK 11857	23.0	23.6	23.9	1.5	1.8
